# 

**DOI:** 10.1093/jlb/lsv009

**Published:** 2015-03-27

**Authors:** Owen D. Jones, Jeffrey D. Schall, Francis X. Shen

When I served as a US District Court Judge for the District of Massachusetts (from 1994 to 2011), I was obliged to instruct the jurors about the law, including, in a criminal case, the legal framework for determining the defendant's state of mind. Criminal offenses typically involve an act which the defendant was alleged to have done, coupled with a culpable mental state—such as ‘an intent to kill’ or ‘premeditation’. The instruction I would give was one which had been approved by the appellate courts, namely:

Ladies and gentlemen of the jury, I want to explain something about evidence of a defendant's state of mind. Ordinarily, there is no way that a defendant's state of mind can be proved directly *because no one can read another person's mind and tell what that person is thinking.*

Nevertheless, I would tell them, that they could infer a defendant's state of mind from the surrounding circumstances, what we called circumstantial evidence. And I would add: ‘That evidence might include what the defendant said, what the defendant did, how the defendant acted, and “any other facts or circumstances in evidence that show what was in the defendant's mind”’.

*Law and Neuroscience* raises the fascinating question—among many, many others: What if neuroscientists purported to be able to ‘read another person's mind’ to some degree, or at the very least, to offer more direct data on a person's mental state then jurors and judges have been able to consider before? Should such evidence be admissible in that residual category of ‘any other facts or circumstances…that show what was in the defendant's mind’? What threshold test should we require before we deem it admissible? What limitations? What are the risks of a juror misperceiving the evidence—particularly now, when many of the neuroscience tools are still crude? Will judges and jurors overvalue the evidence, distort its salience beyond what the science justifies? And far beyond admissibility, how far does this data go to challenge fundamental assumptions about agency and responsibility? More important perhaps, how far *should it* go in upending traditional normative judgments about culpability? Is it true, as Jeffrey Rosen indicated: ‘As we learn more about criminals’ brains will we have to redefine our most basic ideas of justice’?

The project that the authors of *Law and Neuroscience* have set about to do could not have been more daunting. It is, after all, the first text on law and neuroscience, providing the reader with a primer in brain science and identifying the areas in which it intersects with legal frameworks. It is a text that seeks to reach a very disparate audience—students, judges, litigators, legislators, and the general public. The book presumes neither familiarity with neuroscience, nor even familiarity with the law. As such, the volume has to operate on several levels at once: It has to introduce the reader to the brain sciences, and their applications. Understanding these applications necessarily involves understanding the fields of law to which the science relates, the essential legal concepts comprising them. And to enable a critical perspective on the part of the reader, which the authors clearly seek to do, the book offers more than just mounds of information. It constantly raises important questions, on a host of controversial subjects, and frames both sides of the debate. In effect, the book offers a platform to start a very important discussion in that it cannot possibly finish. For those who wish to go further, it offers a bibliography, a web site with supplementary materials, and better yet, the promise of future editions.

The materials the authors have selected range from articles in the popular press, to excerpts of scientific papers, judicial opinions (published and unpublished) and even law review articles, selected not just for their relevance to the topic but also for their clarity. The authors’ extremely helpful commentary is found throughout, at the beginning of each part with a general overview, introducing each chapter with a general summary and commentary, and ending with probing transitional notes to the next chapter. Throughout the authors remind the readers of both the ‘sweeping possibilities’ of neuroscience and its ‘constraining realities’:

On the one hand, the ability of new technologies to reveal information about brain structure and brain function, through non-invasive means, is an important step forward in understanding the relationship between brain and behavior, and between human cognition and the human condition. On the other hand, not even the longest technological stride can take us very far down the branching roads of legal complexity where social constructs like “responsibility” reside, and where tradeoffs between the ideals of individualized justice and the realities of imperfect information force hard choices that science alone cannot resolve.

The book is divided into five parts: Part I is an introduction which sweeps widely across the field, highlighting a few of the better known cases in which neuroscience was used, and noting the possibility of future uses. It is an intentionally provocative section, discussing the implications of neurolaw applications, and the constitutional and policy concerns that accompany them. (Can the government require that a defendant submit to a brain scan, just as the government may order DNA testing? While the scan is not physically intrusive or risky, the information it can generate is extraordinarily significant. Does the Fourth Amendment at its core protect more than just searches of homes, private places and papers? Does it protect thoughts and even brain waves? Can an employer require a brain scan as a requirement of employment? What are the privacy implications of such a requirement?)

Part II, ‘Brain, Behavior and Responsibility’ highlights some of the larger issues of the book—the general problems of introducing science into the courtroom, the different premises, goals, and methods of law and science, the problem of group to individual inferences, issues surrounding the admissibility of scientific evidence and of the judge as a gatekeeper. Indeed, does a judge have special responsibilities in addressing neuroscience especially given, what one author described as, its ‘seductive allure’?. The materials on neuroscience and the criminal law are particularly interesting, the extent to which neuroscience raises fundamental questions of agency and free will. And the book's materials on criminal sentencing are particularly helpful. Neuroscience has had special salience as mitigating evidence in capital proceedings; will evidence of impulsivity point in the opposite direction to exacerbate punishment in the interest of preventing future harm. Sentencing, moreover, with its minimal procedural protections and evidentiary rules, raises unique problems of admissibility. Here *Law and Neuroscience* teaches by presenting actual case materials from a criminal sentencing, including a transcript on the admissibility of the evidence (quantitative electroencephalograms), the examination of the relevant experts, and the motions of counsel.

Part III, ‘The Fundamentals of Cognitive Neuroscience’, offers a primer on brain structure and function and the various brain technologies. Consistent with the text's multiple levels, Part III notes the ‘limits and cautions’ of the technologies, again the ‘sweeping possibilities’ and the ‘constraining realities’. (This part, together with the Appendix ‘How to Read a Brain Imaging study’, is especially helpful for students and those new to the field.)

Part IV moves to more specific issues—further elaborating on the themes of Part I: ‘The Injured Brain’ (brain death as a clinical and a legal matter, injury, pain, and distress); ‘The Thinking and Feeling Brain’ (including issues of eyewitness identification and memory, emotions, and lie detection); and ‘The Developing and Addicted Brain’ (dealing with the adolescent brain and drug addiction).

I was particularly intrigued with the material on judging. Chapter 16 (Judging) provides materials on the neuroscience of legal reasoning, as well as the problem of implicit bias and racism. Chapter 14 (Emotions), considers issues concerning the ‘emotional juror’, ‘the emotional judge’, and ‘the emotional legislator’. What is the kind of decision-making that jurors are supposed to bring to the legal process? Is it only rational or should it also be emotional and empathic? Should victim impact statements be admitted in capital proceedings? And in civil cases, what happens when a jury hears in a single proceeding both the evidence of liability and evidence on pain and suffering, which bears on damages? What kind of decision-making should judges engage in? What is the role of experience, empathy and emotional engagement? And, as is the case throughout the book, the authors do not shy away from the implications of the materials they present, however controversial, whether it be the section on fetal pain, or on the policy implications of the neurobiology of addiction.

Finally, part V introduces the reader to what can be variously described as the wonderful possibilities or the brave new world of neuroscience—cognitive enhancement, manipulating memory, drugs and machines, and artificial intelligence. Here again, the materials are presented in a way that not only edifies, but enables a critical perspective. What are the legal implications of the brain–machine interface devices for privacy, for personal autonomy?

This is not the first time that time worn legal concepts have been challenged by an evolving science and technology. But rarely does one see a volume as comprehensive as this so early in the discussion of neurolaw, raising many questions before they hit the courts or legislatures, before the law responds in inconsistent directions, before practices are written in stone. It is an invaluable book on many levels to many constituencies—students, professors, scholars, lawyers, and not the least of which, judges.

**The Honorable Nancy Gertner****(Retired)**

